# The complete plastome of *Taxillus vestitus* (Loranthaceae), a hemiparasitic plant

**DOI:** 10.1080/23802359.2019.1667912

**Published:** 2019-09-23

**Authors:** Xiaorong Guo, Zhijie Ruan, Guangfei Zhang

**Affiliations:** a Institute of Ecology and Geobotany, Yunnan University, Kunming, Yunnan, China;; b School of Ecology and Environmental Science, Yunnan University, Kunming, Yunnan, China

**Keywords:** Stem hemiparasite, plastome, *Taxillus vestitus*, Loranthaceae, Santalales

## Abstract

*Taxillus vestitus* (Wallich) Danser (Loranthaceae) is a stem hemiparasite occurring in southwest China and Himalayas. In this study, we report the first complete plastome of this species. The plastome is 122,200 bp in size, which encodes 94 unique genes (64 protein-encoding gens, 4 ribosomal RNAs, and 26 tRNAs). Compared to the plastomes of the autotrophic relatives, the *T. vestitus* plastome is significantly reduced. A total of 15 protein-coding genes, and 4 tRNAs were deleted from the plastome.

The lifestyle transition from autotrophy to parasitism leads to varying degrees of plastome reduction (Wicke and Naumann 2018). There are ∼4500 parasitic species documented in 20 angiosperm families, which can be further divided into hemiparasites and holoparasites (Heide-Jørgensen 2008). Hemiparasitic plants, which are capable of photosynthesis, comprise more than 90% of plant parasites (The Parasitic Plant Connection 1997). Unfortunately, the plastomes of only a small fraction of hemiparasites have been sequenced, their plastomes thus remain poorly characterized.

Here, we report the complete plastome sequence of *Taxillus vestitus* (Wallich) Danser, a hemiparasitic shrub of the family Loranthaceae A. L. Jussieu. This species, occurring in southwest China and Himalayas, often parasitizes on the stem of oaks (Qiu and Gilbert 2003). Specimen and leaf tissues of *T. vestitus* were collected from Yunlong, Yunnan, China (N25unnan, China (China (long, Voucher (GF Zhang 2018003) was identified by Dr. Yunheng Ji and deposited at the herbarium of Kunming Institute of Botany, Chinese Academy of Science (KUN). Genomic DNA was extracted from silica gel dried leaves for preparation of sequencing library. Shotgun sequencing was performed on a Illumina HiSeq 2500 platform (Illumina, San Diego, CA). Illumina reads was assembled into plastome using CLC genome assembler v. 4.0β (CLC Inc., Aarhus, Denmark) with default parameter setting, using the plastome of *Taxillus chinensis* (Candolle) Danser (KY996492) as reference. Plastome annotation was performed using Dual Organellar Genome Annotator database (Wyman et al. 2004). The protein-coding genes with one or more frameshift mutations or premature stop codons were annotated as pseudogenes. Start and stop codons and intron/exon boundaries for protein-coding genes were checked manually. Transfer RNA (tRNA) genes were further verified by tRNAscan-SE 1.21 (Schattner et al. 2005) with the default parameters. Plastome sequence of *T. vestitus* was deposited in the NCBI GenBank database (MN175257).

The *T. vestitus* plastome is 122,200 bp in size, and presents a typical quadripartite structure consisting of a large single-copy region (70,250 bp), a small single-copy region (6104 bp), and a pair of inverted repeat regions (22,923 bp each). It encodes 94 unique genes, including 64 protein-encoding gens, 4 ribosomal RNAs, and 26 tRNAs. Compared with plastomes of the majority autotrophic angiosperms that encode 113 unique genes (Wicke and Naumann 2018), a total of 19 genes were deleted from the *T. vestitus* plastome, including 15 protein-coding genes (all the plastid *ndh* genes [11], *rps*15, *rps*16, *rpl*32, and *inf*A), and 4 tNNAs (*trn*G-UCC, *trn*H-GUG, *trn*K-UUU, and *trn*V-UAC).

The relationships of *T. vestitus* with other Loranthaceae species (Figure 1) were reconstructed based on complete plastome DNA sequences, using standard maximum likelihood (ML) method. The plastome of *Erythropalum scandens* Blume (NC_036759) was used as the outgroup to root the tree. Sequences were aligned using MAFFT (Kazutaka and Standley 2013). ML analysis was conducted using RAxML-HPC BlackBox v8.1.24 (Stamatakis 2006) with 1000 replicates of rapid bootstrapping (BS) under the GTRGAMMAI model. Phylogenetic analysis resolved *Taxillus* Tieghem as a monophyletic unit (BS = 100%). The sister relationship between *T. vestitus* and the clade comprising of *Taxillus nigrans* (Hance) Danser and *Taxillus sutchuenensis* (Lecomte) Danser was recovered (BS = 100%).

**Figure 1.**
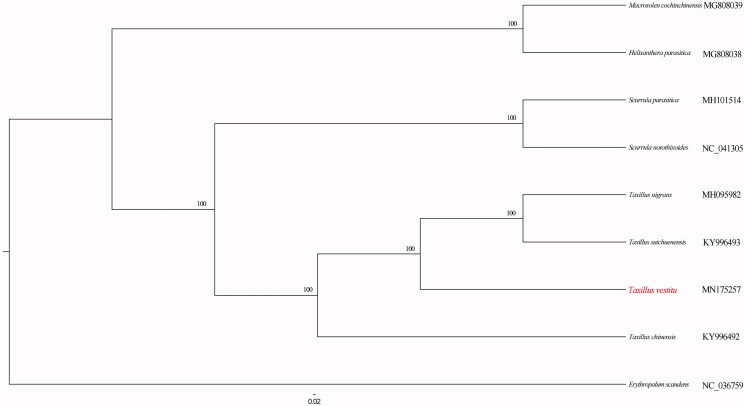
Phylogenetic relationships of *Taxillus vestitus* with other Loranthaceae species. The number on each node indicated bootstrap percentage.
